# Genome resequencing data for Iranian local dogs and wolves

**DOI:** 10.1186/s13104-020-05271-3

**Published:** 2020-09-16

**Authors:** Zeinab Amiri Ghanatsaman, Guo-Dong Wang, Masood Asadi Fozi, Ya-Ping Zhang, Ali Esmailizadeh

**Affiliations:** 1 Department of Animal Science, Faculty of Agriculture, Shahid Bahonar University of Kerman, PB 76169-133 Kerman, Iran; 2 Young Researchers Society, Shahid Bahonar University of Kerman, PB 76169-133 Kerman, Iran; 3 State Key Laboratory of Genetic Resources and Evolution, Kunming Institute of Zoology, Chinese Academy of Sciences, No. 32 Jiaochang Donglu, Kunming, 650223 Yunnan China; 4 State Key Laboratory for Conservation and Utilization of Bio-Resources in Yunnan, Yunnan University, Kunming, 650091 China

**Keywords:** Whole-genome resequencing, Canid, Iran

## Abstract

**Objective:**

The data provided herein represent the whole-genome resequencing data related to three wolves and three Iranian local dogs. The understanding of genome evolution during animal domestication is an interesting subject in genome biology. Dog is an excellent model for understanding of domestication due to its considerable variety of behavioral and physical traits. The Zagros area of current day Iran has been identified as one of the initial centers of animal domestication. The availability of the complete genome sequences of Iranian local canids can be a valuable resource for researchers to address questions and testing hypotheses on the dog domestication process.

**Data description:**

We collected blood samples from six Iranian local canids including two hunting dogs (Saluki breed), a mastiff dog (Qahderijani ecotype) and three wolves. We extracted genomic DNA from blood samples. Sequence data were produced using the Illumina HiSeq 2500 system. All sequence data are available in the National Genomics Data Center (NGDC), Genome Sequence Archive (GSA) database under the accession of CRA001324 and the National Center for Biotechnology Information (NCBI) under the accession of PRJNA639312. The short-read sequences with the mean depth of 16X were aligned to the dog reference genome (CanFam3.1) and achieved 99% coverage of the reference assembly. The obtained information from this experiment will be useful in evolutionary biology.

## Objective

Dogs (*Canis familiaris*) were probably the earliest domesticated animals and one of the human companions in ancient times [1, 2]. Archaeological findings and genetic research indicated that the dog breeds have derived from wild wolves [3–5]. In the Southwest Asia, major–scale farming extended within the so-named Fertile Crescent (FC), where the independent domestication of plants and animals occurred [6, 7]. Extensively, cultural advances occurred in the Zagros area of current day Iraq and Iran, connecting Iranian plateau and Mesopotamia [8]. Dogs had been pictured frequently in Southwest Asia [1, 9]. Consequently, one of the notable viewpoints on the primary location of the dog domestication has been the Southwest Asia, likely the Middle East [1]. Moreover, the Middle East has been included in the considerable allelic distribution between dog breeds and wolf [10]; however, this presumption has been queried because of dog-wolf hybridization as stated in previous studies [11–13]. The dog is a considerable example of phenotypic variation under artificial selection and demographic forces, but genetic basis of this diversity is not yet completely clear. Therefore, the availability of complete whole-genome resequencing data of Iranian local canids will provide an opportunity for researchers to trace the origin of dog domestication. We firstly carried out genome sequencing of six Iranian local canids including two hunting dogs (Saluki breed), a mastiff dog (Qahderijani ecotype) and three wolves (Table 1). We used these data for identifying effective genomic variants in dogs and wolves [14].

**Table 1:** Overview of whole-genome sequence data files of six Iranian canids

Label	Name of data file/data set	File types(extension)	Data repository and identifier (DOI or accession number)
Bioproject [24]	Whole genome resequencing of the Iranian native dogs and wolves	No file type	PRJCA00118 https://bigd.big.ac.cn/bioproject/browse/PRJCA001183
Data file 1 [25–28]	YPi2985_L4_1_clean.fq.gz YPi2985_L4_2_clean.fq.gz YPi2985_L5_1_clean.fq.gz YPi2985_L5_2_clean.fq.gz YPi2985_L7_1_clean.fq.gz YPi2985_L7_2_clean.fq.gz YPi2985_L8_1_clean.fq.gz YPi2985_L8_2_clean.fq.g	FASTQ (fq.gz)	NGDC, Genome Sequence Archive https://bigd.big.ac.cn/gsa/browse/CRA001324/CRR042720 NGDC, Genome Sequence Archive https://bigd.big.ac.cn/gsa/browse/CRA001324/CRR042721 NGDC, Genome Sequence Archive https://bigd.big.ac.cn/gsa/browse/CRA001324/CRR042722 NGDC, Genome Sequence Archive https://bigd.big.ac.cn/gsa/browse/CRA001324/CRR042723
Data file 2 [29–31]	b_L3_1_clean.fq.gz b_L3_2_clean.fq.gz b_L4_1_clean.fq.gz b_L4_2_clean.fq.gz b_L6_1_clean.fq.gz b_L6_2_clean.fq.gz	FASTQ (fq.gz)	NGDC, Genome Sequence Archive https://bigd.big.ac.cn/gsa/browse/CRA001324/CRR042724 NGDC, Genome Sequence Archive https://bigd.big.ac.cn/gsa/browse/CRA001324/CRR042725 NGDC, Genome Sequence Archive https://bigd.big.ac.cn/gsa/browse/CRA001324/CRR042726
Data file 3 [32–34]	8-a_L5_1_clean.fq.gz 8-a_L5_2_clean.fq.gz 8-a_L6_1_clean.fq.gz 8-a_L6_2_clean.fq.gz 8-a_L8_1_clean.fq.gz 8-a_L8_2_clean.fq.gz	FASTQ (fq.gz)	NGDC, Genome Sequence Archive https://bigd.big.ac.cn/gsa/browse/CRA001324/CRR042727 NGDC, Genome Sequence Archive https://bigd.big.ac.cn/gsa/browse/CRA001324/CRR042728 NGDC, Genome Sequence Archive https://bigd.big.ac.cn/gsa/browse/CRA001324/CRR042729
Data file 4 [35–37]	74_L1_1_clean.fq.gz 74_L1_2_clean.fq.gz 74_L5_1_clean.fq.gz 74_L5_2_clean.fq.gz 74_L8_1_clean.fq.gz 74_L8_2_clean.fq.gz	FASTQ (fq.gz)	NGDC, Genome Sequence Archive https://bigd.big.ac.cn/gsa/browse/CRA001324/CRR042730 NGDC, Genome Sequence Archive https://bigd.big.ac.cn/gsa/browse/CRA001324/CRR042731 NGDC, Genome Sequence Archive https://bigd.big.ac.cn/gsa/browse/CRA001324/CRR042732
Data file 5 [38–41]	85_L5_1_clean.fq.gz 85_L5_2_clean.fq.gz 85_L6_1_clean.fq.gz 85_L6_2_clean.fq.gz 85_L7_1_clean.fq.gz 85_L7_2_clean.fq.gz 85_L8_1_clean.fq.gz 85_L8_2_clean.fq.gz	FASTQ (fq.gz)	NGDC, Genome Sequence Archive https://bigd.big.ac.cn/gsa/browse/CRA001324/CRR042733 NGDC, Genome Sequence Archive https://bigd.big.ac.cn/gsa/browse/CRA001324/CRR042734 NGDC, Genome Sequence Archive https://bigd.big.ac.cn/gsa/browse/CRA001324/CRR042735 NGDC, Genome Sequence Archive https://bigd.big.ac.cn/gsa/browse/CRA001324/CRR042736
Data file 6 [42–45]	1 _L1_1_clean.fq.gz 1_L1_2_clean.fq.gz 1_L2_1_clean.fq.gz 1_L2_2_clean.fq.gz 1_L3_1_clean.fq.gz 1_L3_2_clean.fq.gz 1_L4_1_clean.fq.gz 1_L4_2_clean.fq.gz	FASTQ (fq.gz)	NGDC, Genome Sequence Archive https://bigd.big.ac.cn/gsa/browse/CRA001324/CRR042737 NGDC, Genome Sequence Archive https://bigd.big.ac.cn/gsa/browse/CRA001324/CRR042738 NGDC, Genome Sequence Archive https://bigd.big.ac.cn/gsa/browse/CRA001324/CRR042739 NGDC, Genome Sequence Archive https://bigd.big.ac.cn/gsa/browse/CRA001324/CRR042740

## Data description

We collected blood samples from three Iranian local dogs and three Iranian local wolves with the approval of the owners from six various sites in Iran. Sampling of Saluki dogs was done on Jamil Tavanaei’s personal farms in Kurdistan zone (Sanandaj and Bijar) and sampling of a Qahderijani dog was conducted on Alireza Hoseini private farm in Isfahan zone. One of the wolf samples was collected from Kerman zoological garden in Kerman zone and the others were collected from Eram zoological garden in Tehran zone. DNA was extracted with phenol/chloroform method. For sequencing library preparation, the genomic DNA was sheared to fragments of 300–500 bp, which were then end-repaired, “A”-tailed, and ligated to Illumina sequencing adapters. The ligated products with sizes of 400–500 bp were selected on 2% agarose gels and then amplified by LM-PCR. Illumina paired-end whole-genome resequencing for six individuals was done with Hiseq2500 Illumina system) http://www.berrygenomics.com). Both nuclear and mitochondrial genomes were sequenced. We created 287.5 Gb data with a uniform read length of 150 bp. A total of 1,884,054,828 short reads were generated for all of the six individuals. After filtering, the range of total high-quality sequence data was from 42.1 Gb to 51 Gb and the coverage varied from 14.51X to 17.15X. The range of the mean insert sizes and their standard deviations in the sequenced data for all samples was from 280.06 to 331.86 and from 27.12 to 33.94, respectively.

The quality assessment of raw sequence reads was done with FastQC (http://www.bioinformatics.babraham.ac.uk/projects/fastqc/). We used BWA (v.0.7.15) [15] program to compare sequence data with the reference genome (CanFam3.1) downloaded from the Ensembl (http://asia.ensembl.org/Canis_lupus_familiaris/Info/Index). The alignment quality was assessed with SAMtools v.1.9 using flagstat and depth commands [16]. The short-read sequences with the mean depth of 16X were mapped to the dog reference genome (CanFam3.1) and achieved 99% coverage of the reference assembly. The mapping output files were preprocessed using SAMtools [16], the Picard tools (http://broadinstitute.github.io/picard/) and GATK tools [17]. We used variome detection pipeline for this data using CNVnator [18], BreakDancer [19], DELLY [20] and Bedtools [21] programs [14]. Finally, we compared the effect of variome between the dog and wolf genomes using Sorting Intolerant from Tolerant (SIFT) algorithm [19], Ensembl annotation [22] and DAVID [23] tool [14]. The data presented herein together with our previously mitochondrial DNA sequence on Iranian dogs [11] will provide useful resources to understand genetic structure of the Iranian dogs and testing hypotheses on the dog origin and domestication issues.

## Limitations

Sample size for the dog and wolf populations is a limitation of our work. We could create genome sequence data from only three wolves and three dogs. In addition, we produced the short-reads with a mean depth of 16X which is a medium depth and it might not be suitable for some genomic analyses.

## Data Availability

The raw data reported here are available in the NGDC, GSA database (https://bigd.big.ac.cn/gsa/) under the accession number of CRA001324 and NCBI under the accession of PRJNA639312. Please see the data files 1 to 6 in Table 1 for more details on the raw sequence data [24–45].

## Abbreviations

FC: Fertile crescent
GSA: Genome sequence archive
NCBI: National Center for Biotechnology Information
NGDC: National Genomics Data Center
SIFT: Sorting intolerant from tolerant

## References

[1] Clutton-Brock J (1981). Domesticated animals: from early times, Heinemann in assoc. with British Museum.

[2] Wang GD, Zhai W, Yang HC, Wang L, Zhong L, Liu YH (2016). Out of southern East Asia: the natural history of domestic dogs across the world. Cell Res.

[3] Clutton-Brock J, Serpell J (1995). Origins of the dog: domestication and early history. The domestic dog: its evolution, behaviour, and interactions with people.

[4] Vilà C, Savolainen P, Maldonado JE, Amorim IR, Rice JE, Honeycutt RL (1997). Multiple and ancient origins of the domestic dog. Science.

[5] Wayne RK (1993). Molecular evolution of the dog family. Trends Genet.

[6] Colledge S, Conolly J, Shennan S, Bellwood P, Bouby L, Hansen J (2004). Archaeobotanical evidence for the spread of farming in the Eastern Mediterranean 1. Curr Anthropol..

[7] Zeder MA (2008). Domestication and early agriculture in the Mediterranean Basin: origins, diffusion, and impact. Proc Natl Acad Sci U S A..

[8] Alizadeh A (2010). The rise of the highland Elamite state in southwestern Iran. Curr Anthropol..

[9] Przezdziecki XJB, Paris G (2001). Our levriers: the past, present and future of all sighthounds.

[10] VonHoldt BM (2010). Genome-wide SNP and haplotype analyses reveal a rich history underlying dog domestication. Nature.

[11] Amiri Ghanatsaman Z, Adeola AC, Asadi Fozi M, Ma YP, Peng MS, Wang GD (2017). Mitochondrial DNA sequence variation in Iranian native dogs. Mitochondrial DNA A DNA Mapp Seq Anal..

[12] Ardalan A, Kluetsch CF, Zhang AB, Erdogan M, Uhlén M, Houshmand M (2011). Comprehensive study of mtDNA among Southwest Asian dogs contradicts independent domestication of wolf, but implies dog–wolf hybridization. Ecol Evol..

[13] Freedman AH, Gronau I, Schweizer RM, Ortega-Del Vecchyo D, Han E, Silva PM (2014). Genome sequencing highlights the dynamic early history of dogs. PLoS Genet.

[14] Amiri Ghanatsaman Z, Wang GD, Asadollahpour Nanaei H, Asadi Fozi M, Peng MS, Esmailizadeh A (2020). Whole genome resequencing of the Iranian native dogs and wolves to unravel variome during dog domestication. BMC Genomics..

[15] Li H, Durbin R (2009). Fast and accurate short read alignment with burrows–wheeler transform. Bioinformatics.

[16] Li H, Handsaker B, Wysoker A, Fennell T, Ruan J, Homer N (2009). The sequence alignment/map format and SAMtools. Bioinformatics.

[17] McKenna A, Hanna M, Banks E, Sivachenko A, Cibulskis K, Kernytsky A (2010). The genome analysis toolkit: a MapReduce frame work for analyzing nextgeneration DNA sequencing data. Genome Res.

[18] Abyzov A, Urban AE, Snyder M, Gerstein M (2011). CNVnator: an approach to discover, genotype, and characterize typical and atypical CNVs from family and population genome sequencing. Genome Res.

[19] Chen K, Wallis J, McLellan M (2009). BreakDancer: an algorithm for high-resolution mapping of genomic structural variation. Nat Methods.

[20] Rausch T, Zichner T, Schlattl A, Stütz AM, Benes V, Korbel JO (2012). DELLY: structural variant discovery by integrated paired-end and split-read analysis. Bioinformatics.

[21] Quinlan AR, Hall IM (2010). BEDTools: a flexible suite of utilities for comparing genomic features. Bioinformatics.

[22] Flicek P, Amode MR, Barrell D, Beal K, Brent S, Carvalho-Silva D (2011). Ensembl 2012. Nucleic Acids Res.

[23] Huang DW, Sherman BT, Lempicki RA (2009). Systematic and integrative analysis of large gene lists using DAVID bioinformatics resources. Nat Protoc.

[24] BIGD Genome Warehouse; 2020. https://bigd.big.ac.cn/bioproject/browse/PRJCA001183.

[25] BIGD Genome Warehouse; 2020. https://bigd.big.ac.cn/gsa/browse/CRA001324/CRR042720.

[26] BIGD Genome Warehouse; 2020. https://bigd.big.ac.cn/gsa/browse/CRA001324/CRR042721.

[27] BIGD Genome Warehouse; 2020. https://bigd.big.ac.cn/gsa/browse/CRA001324/CRR042722.

[28] BIGD Genome Warehouse; 2020. https://bigd.big.ac.cn/gsa/browse/CRA001324/CRR042723.

[29] BIGD Genome Warehouse; 2020. https://bigd.big.ac.cn/gsa/browse/CRA001324/CRR042724.

[30] BIGD Genome Warehouse; 2020. https://bigd.big.ac.cn/gsa/browse/CRA001324/CRR042725.

[31] BIGD Genome Warehouse; 2020. https://bigd.big.ac.cn/gsa/browse/CRA001324/CRR042726.

[32] BIGD Genome Warehouse; 2020. https://bigd.big.ac.cn/gsa/browse/CRA001324/CRR042727.

[33] BIGD Genome Warehouse; 2020. https://bigd.big.ac.cn/gsa/browse/CRA001324/CRR042728.

[34] BIGD Genome Warehouse; 2020. https://bigd.big.ac.cn/gsa/browse/CRA001324/CRR042729.

[35] BIGD Genome Warehouse; 2020. https://bigd.big.ac.cn/gsa/browse/CRA001324/CRR042730.

[36] BIGD Genome Warehouse; 2020. https://bigd.big.ac.cn/gsa/browse/CRA001324/CRR042731.

[37] BIGD Genome Warehouse; 2020. https://bigd.big.ac.cn/gsa/browse/CRA001324/CRR042732.

[38] BIGD Genome Warehouse; 2020. https://bigd.big.ac.cn/gsa/browse/CRA001324/CRR042733.

[39] BIGD Genome Warehouse; 2020. https://bigd.big.ac.cn/gsa/browse/CRA001324/CRR042734.

[40] BIGD Genome Warehouse; 2020. https://bigd.big.ac.cn/gsa/browse/CRA001324/CRR042735.

[41] BIGD Genome Warehouse; 2020. https://bigd.big.ac.cn/gsa/browse/CRA001324/CRR042736.

[42] BIGD Genome Warehouse; 2020. https://bigd.big.ac.cn/gsa/browse/CRA001324/CRR042737.

[43] BIGD Genome Warehouse; 2020. https://bigd.big.ac.cn/gsa/browse/CRA001324/CRR042738.

[44] BIGD Genome Warehouse; 2020. https://bigd.big.ac.cn/gsa/browse/CRA001324/CRR042739.

[45] BIGD Genome Warehouse; 2020. https://bigd.big.ac.cn/gsa/browse/CRA001324/CRR042740.

